# Clinicopathological Characteristics of Breast Cancer in Women Aged 40 Years or Younger in Saudi Arabia: A Single-Center Descriptive Study

**DOI:** 10.3390/jcm15218293

**Published:** 2026-10-24

**Authors:** Nada A. Alyousefi, Leen M. Almaghyuli, Sarah A. Alsultan, Norah A. Alshehri, Ambreen Kazi, Lemmese Alwatban

**Affiliations:** 1 Department of Family and Community Medicine, College of Medicine, King Saud University, P.O. Box 2925, Riyadh 11461, Saudi Arabia; nalyousefi@ksu.edu.sa (N.A.A.); drnora@ksu.edu.sa (N.A.A.); lalwatban@ksu.edu.sa (L.A.); 2 University Family Medicine Center, King Saud University Medical City, King Saud University, Riyadh 11495, Saudi Arabia; leen.majed19@gmail.com; 3 Department of Radiology and Medical Imaging, King Saud University Medical City, King Saud University, P.O. Box 7805, Riyadh 11472, Saudi Arabia; salsultana@ksu.edu.sa; 4 Women’s Health Research Chair, Deanship of Research Chairs, King Saud University, P.O. Box 230520, Riyadh 11321, Saudi Arabia

**Keywords:** breast cancer, young women, clinicopathological characteristics, molecular subtype, Saudi Arabia

## Abstract

**Background/Objectives:** Breast cancer is the most commonly diagnosed cancer among women in Saudi Arabia, yet evidence on younger women remains limited. This study aimed to characterize the clinicopathological, molecular, and treatment features of women diagnosed at age ≤ 40 years and to explore differences between women aged ≤ 35 and 36–40 years. **Methods:** We conducted a retrospective study of 135 women aged 22–40 years diagnosed with breast cancer at King Saud University Medical City, Riyadh. Variable-specific available-case denominators were used without imputation. Characteristics were summarized descriptively, and age groups were compared using Pearson’s chi-square or exact tests, as appropriate. **Results:** A breast mass was the most frequent presentation (79.3%), and invasive ductal carcinoma was the predominant diagnosis (80.7%). Tumors > 2 to ≤5 cm accounted for 48.9% of cases; axillary involvement was documented in 47.4%, distant metastasis in 16.7%, and grade 3 disease in 68.2% of patients with documented grade. Luminal B (31.5%) and triple-negative (29.2%) were the most frequently documented molecular subtypes. No statistically significant differences were detected between the age groups in clinical presentation, tumor-size category, axillary involvement, distant metastasis, histological grade, documented molecular subtype, or treatment characteristics. **Conclusions:** Symptomatic presentation and potentially unfavorable pathological features were common in this tertiary-center cohort. The absence of statistically significant clinicopathological, molecular, or treatment differences between age groups does not establish clinical equivalence and should be interpreted cautiously given the exploratory design and modest sample size. The findings highlight the need for standardized staging, hereditary-risk assessment, and multicenter longitudinal data to inform care for younger women with breast cancer.

## 1. Introduction

Breast cancer was the most frequently diagnosed cancer among women worldwide in 2022, accounting for approximately 2.3 million new cases and 666,000 deaths [1]. In Saudi Arabia, it remains the most commonly diagnosed cancer among women, and registry analyses indicate that its burden has increased over recent decades [2,3,4,5]. According to the Saudi Cancer Incidence Report 2024, 4601 breast cancer cases were registered among Saudi females, accounting for 34.8% of all cancers diagnosed in Saudi women [4]. The age-standardized incidence rate was 63.3 per 100,000 Saudi females, and the median age at diagnosis was 53 years [4]. Although the age-specific incidence rate increased with age and peaked among women aged 60–64 years, breast cancer accounted for 39.7% of all cancers diagnosed among Saudi women aged 30–44 years [4]. These national data demonstrate the substantial burden of breast cancer among Saudi women and support focused investigation of younger patients.

Breast cancer diagnosed at 40 years of age or younger represents a clinically important subgroup. International consensus guidance commonly defines breast cancer in young women as breast cancer diagnosed at age ≤ 40 years, whereas some studies use ≤35 years to distinguish a “very young” subgroup [6,7]. Breast cancer in younger women may be associated with distinct clinical challenges, including potentially aggressive tumor characteristics, fertility considerations, premature menopause, genetic counseling needs, and age-specific psychosocial and survivorship concerns [6,8,9]. Nevertheless, younger women remain underrepresented in many breast cancer studies, and evidence concerning their clinicopathological characteristics varies across populations and healthcare settings.

Published cohorts indicate that young women with breast cancer commonly present symptomatically, particularly with a palpable breast mass, and that invasive ductal carcinoma is the predominant histological type [9,10]. Tumor size, histological grade, lymph-node involvement, hormone-receptor status, human epidermal growth factor receptor 2 status, and documented molecular subtype are clinically important because they inform prognosis and treatment planning [7,9,11,12,13,14]. However, these findings have not been consistent across all studies.

Regional studies have similarly described symptomatic presentation and potentially unfavorable pathological characteristics among young women with breast cancer. A retrospective study from Egypt involving women younger than 35 years reported frequent axillary lymph-node involvement and a substantial proportion of estrogen-receptor-negative tumors [7]. Studies from Jordan and Saudi Arabia have also documented heterogeneity in tumor characteristics, stage at presentation, molecular subtypes, treatment patterns, and clinical outcomes among women younger than 40 years [11,12,14,15]. Differences among these studies may reflect variations in study populations, referral patterns, diagnostic practices, institutional case mix, study periods, and the age thresholds used to define young and very young women.

Despite the substantial burden of breast cancer in Saudi Arabia, detailed data describing the demographic, clinicopathological, molecular, and treatment characteristics of women diagnosed at age ≤ 40 years remain limited. Age-related variation within this young population is also not well characterized. Institution-based data cannot estimate population incidence or determine causal risk factors, but they can provide detailed clinical characterization and identify priorities for larger population-based and multicenter studies.

Accordingly, the primary aim of this study was to describe the demographic, clinical, pathological, documented molecular, and treatment characteristics of women diagnosed with breast cancer at age ≤ 40 years at a tertiary-care center in Saudi Arabia. A secondary exploratory objective was to compare these characteristics between women aged ≤ 35 years and those aged 36–40 years.

## 2. Materials and Methods

### 2.1. Study Design and Setting

This retrospective observational study was conducted at King Saud University Medical City, a tertiary-care center and teaching hospital in Riyadh, Saudi Arabia. The study was designed as a descriptive clinical epidemiological analysis of women diagnosed with breast cancer at age ≤ 40 years, with an exploratory comparison between women aged ≤ 35 years and those aged 36–40 years.

### 2.2. Study Period and Population

The study included eligible breast cancer records dated from 2016 through 2024. The retrospective review and data extraction were conducted during 2023–2024 using records available in the institutional electronic health record and radiology databases. Eligible cases were female patients aged ≤ 40 years identified in the institutional database using a predefined set of diagnostic terms and keywords related to breast cancer, without restriction by histological type. Men with breast cancer and women diagnosed after age 40 years were excluded.

### 2.3. Sample Size and Selection

**Figure 1:**
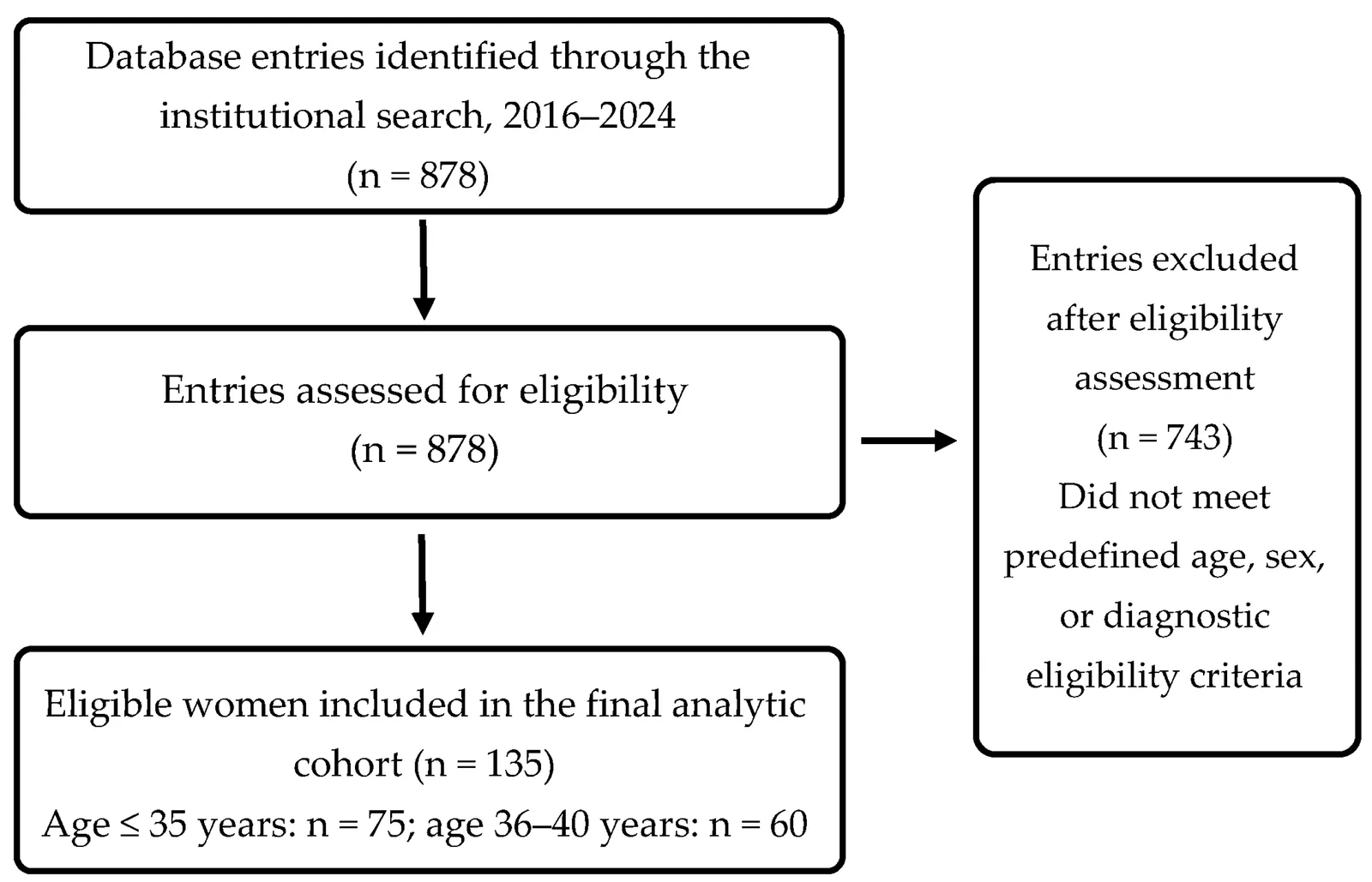
Case-selection flow diagram. Database entries refer to search results reviewed for eligibility.

A census sampling approach was used to include all eligible records identified during data extraction. Because all available eligible records were included, no formal sample-size calculation was performed. The final analytic dataset comprised 135 patients aged 22–40 years. For exploratory age-group comparisons, patients were categorized as very young (≤35 years; *n* = 75) or young (36–40 years; *n* = 60). The ≤35-year threshold was selected for this exploratory comparison because it has been used in prior studies to define a “very young” subgroup and facilitates comparison with the previous literature; it was not intended as a biological cutoff [7]. The case-selection process is summarized in Figure 1.

### 2.4. Data Collection

Following institutional approval, trained data collectors manually extracted information from the King Saud University Medical City electronic health record and radiology databases using a standardized data-collection form. The form was adapted from that used by Cala-Ayala et al. [10]. Permission to use the form was obtained from the corresponding author.

### 2.5. Study Variables and Definitions

The extracted variables included age, marital status, number of children, residence, parity status, family history of breast cancer, body mass index, initial presentation, histological diagnosis, tumor size, axillary involvement, distant metastasis, histological grade, documented molecular subtype, primary treatment, type of surgery, recurrence status, and vital status at the last documented contact. Parity status was recorded as parous or nulliparous according to the available medical-record documentation. Family history of breast cancer was coded as present when it was explicitly documented in the medical record; degree of relationship and relatives’ ages at diagnosis were not consistently available. Screening detection was coded only when the source record explicitly identified screening; the records did not reliably distinguish organized screening, opportunistic screening, high-risk surveillance, or other pathways.

Body mass index was categorized as normal weight (18.5–24.9 kg/m^2^), overweight (25.0–29.9 kg/m^2^), or obesity (≥30.0 kg/m^2^). Tumor size was grouped as ≤2 cm, >2 to ≤5 cm, or >5 cm. These were descriptive size-based groups and should not be interpreted as complete American Joint Committee on Cancer T classifications. Complete AJCC/TNM stage, including T4-defining features and composite clinical or pathological stage, was not consistently documented at a uniform time point and could not be reconstructed reliably.

Documented molecular subtype categories—luminal A, luminal B, human epidermal growth factor receptor 2-enriched, triple-negative, and basal-like—were abstracted as recorded in the original histopathology reports. No centralized pathology review or retrospective reclassification using uniform ER, PR, HER2, and Ki-67 criteria was performed. The dataset did not capture assay platforms or year-specific reporting criteria; therefore, consistency of molecular-subtype definitions across 2016–2024 could not be independently verified.

Patients documented as having transferred care were those who continued follow-up at another hospital or moved to another city. Because subsequent clinical information was unavailable, these patients were excluded from the available-case denominators for outcomes when recurrence or vital status could not be established. Treatment variables reflected the broad management labels documented in the source charts. “Neoadjuvant plus endocrine therapy” indicated documentation of both treatment types and did not necessarily imply concurrent administration. Specific agents, doses, cycles, treatment sequence, radiotherapy, treatment completion, and care delivered after transfer were not consistently available. Follow-up duration could not be calculated reliably because dates of diagnosis, transfer, recurrence, last contact, and death were incomplete or inconsistently recorded.

### 2.6. Statistical Analysis

Statistical analyses were performed using IBM SPSS Statistics, version 26. Continuous variables are presented as mean ± standard deviation and range, and categorical variables as frequencies and percentages. Percentages were calculated using the number of patients with available data for each variable. Missing observations were excluded on a variable-by-variable basis, and no imputation was performed. For age-group comparisons, marital status was grouped as married versus unmarried (single/divorced), histological diagnosis as invasive ductal carcinoma versus other diagnoses, and initial presentation as breast mass versus non-mass presentation. Two cases documented as basal-like were retained in the descriptive analysis but excluded from the age-group molecular-subtype comparison because the available pathology records did not provide sufficient standardized information to assign them reliably to one of the four comparison categories (luminal A, luminal B, HER2-enriched, or triple-negative).

The ≤35-year and 36–40-year groups were compared using Pearson’s chi-square test when expected cell counts were adequate. Fisher’s exact test was used for the recurrence comparison because of sparse expected cell counts, and the Fisher–Freeman–Halton exact test was used for the primary-treatment comparison. Following the significant overall BMI-category comparison, three post hoc pairwise 2 × 2 Pearson chi-square tests were performed, with Holm correction applied across these three comparisons. All other comparisons were exploratory, and no global multiplicity adjustment was applied; therefore, statistically significant findings were considered hypothesis-generating. No multivariable analyses were performed because the study was descriptive and the age-group comparisons were exploratory. No time-to-event or survival analysis was undertaken because standardized follow-up durations and complete event dates were not included in the analytic dataset.

### 2.7. Ethical Considerations

The study protocol was approved by the Institutional Review Board of King Saud University (No. 23/0627/IRB). The study was conducted in accordance with King Saud University ethical requirements and the Declaration of Helsinki. Because of the retrospective design and use of routinely collected medical-record data, the Institutional Review Board waived the requirement for individual informed consent.

## 3. Results

### 3.1. Participant and Demographic Characteristics

The study included 135 women aged 22–40 years, with a mean age of 34.0 ± 4.08 years. Data completeness varied across variables because some information was not documented in the medical records; variable-specific denominators are therefore reported throughout the results and tables. Of the 135 women, 75 (55.6%) were aged ≤ 35 years and 60 (44.4%) were aged 36–40 years. Among participants with available data, 84 of 132 (63.6%) were married, 82 of 127 (64.6%) were parous, and 38 of 130 (29.2%) had a family history of breast cancer. The single-year age distribution is shown in Supplementary Figure S1, and variable-level data availability is summarized in Supplementary Table S1. The mean body mass index was 27.6 ± 6.12 kg/m^2^. Additional demographic and reproductive characteristics are presented in Table 1.

### 3.2. Clinical, Pathological, Molecular, Treatment, and Recorded Outcome Characteristics

Invasive ductal carcinoma was the predominant diagnosis, accounting for 109 cases (80.7%), followed by ductal carcinoma in situ in 16 (11.9%). A breast mass was the most frequent initial presentation, reported in 107 patients (79.3%), whereas eight patients (5.9%) were diagnosed through screening.

Tumors measuring > 2 to ≤5 cm represented the most frequent size group, accounting for 66 cases (48.9%), followed by tumors measuring ≤ 2 cm in 38 (28.1%) and tumors measuring > 5 cm in 31 (23.0%). Axillary involvement was documented in 63 of 133 patients with available data (47.4%), and distant metastasis was documented in 22 of 132 patients (16.7%). Among the 129 patients with documented histological grade, 88 (68.2%) had grade 3 disease.

Documented molecular-subtype information was available for 130 patients. Luminal B was the most frequently documented subtype, occurring in 41 patients (31.5%), followed by triple-negative disease in 38 (29.2%), luminal A in 26 (20.0%), human epidermal growth factor receptor 2-enriched disease in 23 (17.7%), and basal-like disease in two (1.5%).

Primary treatment was documented as neoadjuvant systemic treatment in 68 patients (50.4%) and as neoadjuvant plus endocrine therapy in 42 (31.1%). Mastectomy was the most frequently recorded surgical procedure, performed in 70 patients (51.9%), while 37 (27.4%) underwent breast-conserving surgery.

Among the 122 patients with documented recurrence status, eight (6.6%) had a recorded recurrence. Among the 120 patients with known vital status, 108 (90.0%) were alive at the last documented contact and 12 (10.0%) had a recorded death. These findings are descriptive and should not be interpreted as recurrence or survival rates because follow-up duration was not standardized. The complete clinical, pathological, molecular, treatment, and recorded outcome characteristics are presented in Table 2.

**Table 2:** Clinical, pathological, molecular, treatment, and recorded outcome characteristics.

Characteristic	*n* (%)
**Histological diagnosis**	
Invasive ductal carcinoma	109 (80.7)
Ductal carcinoma in situ	16 (11.9)
Invasive lobular carcinoma	2 (1.5)
Malignant phyllodes tumor	2 (1.5)
Other	6 (4.4)
**Initial presentation**	
Breast mass	107 (79.3)
Pain	8 (5.9)
Incidental finding	3 (2.2)
Screening	8 (5.9)
Other	9 (6.7)
**Tumor size, cm, mean ± SD**	3.69 ± 2.61
Tumor-size group (*n* = 135)	
≤2 cm	38 (28.1)
>2 to ≤5 cm	66 (48.9)
>5 cm	31 (23.0)
**Axillary involvement (*n* = 133)**	
Present	63 (47.4)
Absent	70 (52.6)
**Distant metastasis (*n* = 132)**	
Present	22 (16.7)
Absent	110 (83.3)
**Histological grade (*n* = 129)**	
Grade 1	9 (7.0)
Grade 2	32 (24.8)
Grade 3	88 (68.2)
**Documented molecular subtype (*n* = 130)**	
Luminal A	26 (20.0)
Luminal B	41 (31.5)
HER2-enriched	23 (17.7)
Triple-negative	38 (29.2)
Basal-like	2 (1.5)
**Primary treatment**	
None documented	9 (6.7)
Neoadjuvant systemic treatment	68 (50.4)
Neoadjuvant plus endocrine therapy	42 (31.1)
Endocrine therapy	8 (5.9)
Transfer of care	8 (5.9)
**Surgery**	
No surgery documented	19 (14.1)
Breast-conserving surgery	37 (27.4)
Mastectomy	70 (51.9)
Transfer of care	9 (6.7)
**Recorded recurrence status (*n* = 122)**	
No recorded recurrence	114 (93.4)
Recorded recurrence	8 (6.6)
**Vital status at last documented contact (*n* = 120)**	
Alive	108 (90.0)
Death recorded	12 (10.0)

**Note:** Values are presented as *n* (%) unless otherwise stated. Percentages were calculated using available-case denominators. Tumor size was grouped as ≤2 cm, >2 to ≤5 cm, or >5 cm; these groups should not be interpreted as complete TNM T classifications because T4 features were unavailable. Composite clinical and pathological stage was also unavailable. Molecular subtypes were extracted as documented in pathology reports and were not independently reclassified. The “Other” histological diagnoses comprised one case each of ductal carcinoma not otherwise specified, lobular carcinoma not otherwise specified, myelotic sarcoma as documented in the source record, breast sarcoma, invasive carcinoma with basal-like phenotype, and angiosarcoma. Treatment categories reflect the broad management labels documented in the source records; detailed agents, dosing, sequence, radiotherapy, and treatment completion were not consistently available. Transfer-of-care categories for treatment and surgery are shown descriptively in this table but were excluded from the corresponding age-group comparisons in Table 3. Recurrence and vital status are descriptively recorded outcomes and should not be interpreted as recurrence or survival rates because follow-up duration was not standardized. Patients with undocumented outcomes or transfer of care were excluded from the corresponding outcome denominators. HER2, human epidermal growth factor receptor 2; SD, standard deviation.

**Table 3:** Demographic, clinicopathological, molecular, treatment, and recorded outcome characteristics by age group.

Characteristic	Age Group		*p* Value
	≤35 Years *n* (%) (*n* = 75)	36–40 Years *n* (%) (*n* = 60)	
**Marital status (*n* = 132)**			
Unmarried	35 (47.9)	13 (22.0)	**0.002**
Married	38 (52.1)	46 (78.0)	
**Number of children (*n* = 122)**			
None	34 (50.7)	11 (20.0)	**<0.001**
1–2	18 (26.9)	14 (25.5)	
>2	15 (22.4)	30 (54.5)	
**Residence (*n* = 134)**			
Riyadh	65 (86.7)	49 (83.1)	0.560
Outside Riyadh	10 (13.3)	10 (16.9)	
**Parity status (*n* = 127)**			
Parous	35 (50.7)	47 (81.0)	**<0.001**
Nulliparous	34 (49.3)	11 (19.0)	
**Family history of breast cancer (*n* = 130)**			
No	48 (66.7)	44 (75.9)	0.252
Yes	24 (33.3)	14 (24.1)	
**BMI category (*n* = 135)**			
Normal weight	33 (44.0)	13 (21.7)	**0.018**
Overweight	25 (33.3)	24 (40.0)	
Obesity	17 (22.7)	23 (38.3)	
**Histological diagnosis (*n* = 135)**			
Diagnosis other than invasive ductal carcinoma	15 (20.0)	11 (18.3)	0.807
Invasive ductal carcinoma	60 (80.0)	49 (81.7)	
**Initial presentation (*n* = 135)**			
Non-mass presentation	17 (22.7)	11 (18.3)	0.537
Breast mass	58 (77.3)	49 (81.7)	
**Tumor-size category (*n* = 135)**			
≤2 cm	22 (29.3)	16 (26.7)	0.846
>2 to ≤5 cm	35 (46.7)	31 (51.7)	
>5 cm	18 (24.0)	13 (21.7)	
**Axillary involvement (*n* = 133)**			
Present	33 (44.6)	30 (50.8)	0.473
Absent	41 (55.4)	29 (49.2)	
**Distant metastasis (*n* = 132)**			
Present	11 (15.1)	11 (18.6)	0.584
Absent	62 (84.9)	48 (81.4)	
**Histological grade (*n* = 129)**			
Grade 1	6 (8.3)	3 (5.3)	0.769
Grade 2	17 (23.6)	15 (26.3)	
Grade 3	49 (68.1)	39 (68.4)	
**Documented molecular subtype (*n* = 128)**			
Luminal A	18 (25.0)	8 (14.3)	0.391
Luminal B	21 (29.2)	20 (35.7)	
HER2-enriched	14 (19.4)	9 (16.1)	
Triple-negative	19 (26.4)	19 (33.9)	
**Primary treatment (*n* = 127)**			
None documented	4 (5.8)	5 (8.6)	0.577
Neoadjuvant systemic treatment	34 (49.3)	34 (58.6)	
Neoadjuvant plus endocrine therapy	26 (37.7)	16 (27.6)	
Endocrine therapy	5 (7.2)	3 (5.2)	
**Surgery (*n* = 126)**			
No surgery documented	10 (14.5)	9 (15.8)	0.967
Breast-conserving surgery	20 (29.0)	17 (29.8)	
Mastectomy	39 (56.5)	31 (54.4)	
**Recorded recurrence (*n* = 122)**			
No	62 (93.9)	52 (92.9)	1.000
Yes	4 (6.1)	4 (7.1)	
**Recorded death (*n* = 120)**			
No	59 (92.2)	49 (87.5)	0.393
Yes	5 (7.8)	7 (12.5)	

**Note:** Values are presented as *n* (%) using variable-specific available-case denominators. Pearson’s chi-square test was used unless otherwise specified; Fisher’s exact test was used for recorded recurrence and the Fisher–Freeman–Halton exact test for primary treatment. Two basal-like cases were retained descriptively in Table 2 but excluded from the age-group molecular-subtype comparison. Transfer-of-care cases were excluded from the primary-treatment and surgery comparisons. BMI post hoc comparisons used Holm adjustment (Table S2); no global multiplicity adjustment was applied to the remaining exploratory comparisons. Bold *p* values indicate statistical significance at *p* < 0.05. BMI, body mass index; HER2, human epidermal growth factor receptor 2.

### 3.3. Comparisons Between Age Groups

Women aged ≤ 35 years were more frequently unmarried than those aged 36–40 years (47.9% versus 22.0%; *p* = 0.002). They were also more frequently without children (50.7% versus 20.0%) and less frequently had more than two children (22.4% versus 54.5%; overall *p* < 0.001). Nulliparity was more frequent among women aged ≤ 35 years (49.3% versus 19.0%; *p* < 0.001). Body mass index category also differed between the groups (*p* = 0.018), with normal weight more frequent among women aged ≤ 35 years and obesity more frequent among those aged 36–40 years. Post hoc pairwise comparisons showed that normal weight versus obesity remained statistically significant after Holm correction (unadjusted *p* = 0.006; Holm-adjusted *p* = 0.018), whereas normal weight versus overweight (Holm-adjusted *p* = 0.077) and overweight versus obesity (Holm-adjusted *p* = 0.423) were not statistically significant (Table S2).

No statistically significant differences between the age groups were identified in residence, family history of breast cancer, histological diagnosis, initial presentation, tumor-size group, axillary involvement, distant metastasis, histological grade, documented molecular subtype, primary treatment, surgery, recorded recurrence, or recorded death (all *p* > 0.05). Although the overall molecular-subtype distribution did not differ significantly (*p* = 0.391), luminal A was numerically more frequent among women aged ≤ 35 years than among those aged 36–40 years (25.0% vs 14.3%). These comparisons describe the distribution of characteristics among women already diagnosed with breast cancer and should not be interpreted as identifying etiological risk or protective factors. The complete age-group comparisons are presented in Table 3.

## 4. Discussion

This study describes the demographic, clinicopathological, and treatment characteristics of 135 women diagnosed with breast cancer at age ≤ 40 years at a tertiary-care center in Saudi Arabia, including their documented molecular subtypes. More than half of the cohort were aged ≤ 35 years. Most patients presented with a breast mass; invasive ductal carcinoma was the predominant histological type, and substantial proportions had grade 3 tumors, axillary involvement, or distant metastasis. Luminal B and triple-negative breast cancers were the most frequently documented molecular subtypes. Although the two age groups differed in several demographic and reproductive characteristics, no statistically significant differences were identified in clinical presentation, tumor-size group, grade, axillary involvement, distant metastasis, documented molecular subtype, treatment, surgery, recorded recurrence, or recorded death. These age-group comparisons were exploratory and were not designed as equivalence tests.

Women aged ≤ 35 years constituted 55.6% of the cohort. This proportion reflects the composition of patients treated at a single tertiary-care center and should not be interpreted as the population prevalence or incidence of breast cancer among very young Saudi women. In the present cohort, 79.3% of patients presented with a breast mass, whereas only 5.9% were diagnosed through screening. In Saudi Arabia, routine mammographic screening is recommended for women aged 40–69 years, which may partly explain the low proportion of screening-detected cases in this predominantly younger cohort [16]. However, the source records did not reliably distinguish routine screening, opportunistic screening, high-risk surveillance, or other pathways; therefore, the screening-detected cases could not be attributed to a specific screening pathway.

This finding is consistent with previous studies showing that young women with breast cancer commonly present with palpable breast findings. A Colombian study reported palpable findings in approximately 81% of young women, while a study from Johns Hopkins reported palpable breast findings or noticeable breast changes in 84% of women younger than 40 years [9,10]. The predominance of symptomatic presentation may partly reflect the absence of routine population-based mammographic screening for average-risk women younger than 40 years, as well as the diagnostic challenges associated with breast symptoms in women with relatively dense breast tissue [6,17]. However, the present dataset did not include the interval between symptom onset, initial clinical assessment, and pathological diagnosis. The potential contribution of patient-related or healthcare-related diagnostic delay could therefore not be evaluated. Invasive ductal carcinoma accounted for 80.7% of diagnoses, consistent with its predominance in previous cohorts of young women and in the Saudi Cancer Incidence Report 2024 [4,10].

Several potentially unfavorable pathological characteristics were common in this cohort. Tumors measuring > 2 to ≤5 cm accounted for 48.9% of the cohort, while 23.0% measured > 5 cm. Axillary involvement was documented in 47.4%, distant metastasis in 16.7%, and grade 3 disease in 68.2% of patients with a documented histological grade. These findings are broadly consistent with regional reports describing frequent high-grade disease and nodal involvement among young women. For example, a retrospective study from Mansoura University involving women younger than 35 years reported axillary lymph-node involvement in 75.7%, metastatic presentation in 4.7%, and estrogen-receptor-negative disease in 51% of patients [7].

The documented molecular-subtype distribution demonstrated substantial heterogeneity. Luminal B tumors were the most frequently recorded subtype, accounting for 31.5% of patients with a documented subtype, followed by triple-negative, luminal A, and human epidermal growth factor receptor 2-enriched disease. Triple-negative breast cancer represented 29.2% of patients with a documented molecular subtype. The predominance of luminal B disease is consistent with a recent Saudi retrospective cohort in which luminal B was also the most common molecular subtype among women aged ≤ 40 years, accounting for 40.3% of tumors in that age group [15]. Because subtype labels were abstracted as documented and consistency of reporting criteria across the study period could not be independently verified, changes in pathology reporting practices over time may have influenced the documented molecular-subtype distribution.

Previous studies have suggested that younger women may have a greater frequency of biologically aggressive subtypes than older women [7,9,11,12,13,14,15]. However, the present study did not include women older than 40 years and therefore cannot determine whether triple-negative or other aggressive subtypes were more frequent than in the broader Saudi breast cancer population. Furthermore, molecular subtypes were extracted as documented in pathology reports and could not be independently reclassified using standardized immunohistochemical thresholds.

Despite previous reports suggesting that very young women may have more aggressive clinicopathological characteristics than women diagnosed closer to age 40 [11,12,13,14], we did not detect statistically significant differences between women aged ≤ 35 and 36–40 years in clinical presentation, histological diagnosis, tumor-size group, grade, axillary involvement, distant metastasis, or documented molecular subtype. This finding does not establish clinical equivalence. The modest sample size, narrow five-year separation, and exploratory design may have limited statistical power, and a type II error remains possible. Larger multicenter studies are needed to evaluate clinically meaningful age-related differences within young breast cancer populations.

The two age groups differed significantly in marital status, parity status, number of children, and body mass index category. Women aged ≤ 35 years were more frequently unmarried and nulliparous and more often had no children, whereas overweight and obesity were more common among women aged 36–40 years. These findings are likely influenced by the expected association of age with cumulative reproductive experience and changes in body weight. Because the study included only women who had already been diagnosed with breast cancer and did not include a cancer-free comparison group, these variables represent demographic differences between two age strata within the cohort. They cannot be interpreted as causal, protective, or risk factors for developing breast cancer.

Family history of breast cancer was documented in 29.2% of patients, with no statistically significant difference between the two age groups. A family history may indicate the need for hereditary cancer assessment, but it cannot serve as a substitute for genetic testing. Because family history was derived from routine chart documentation rather than a standardized pedigree assessment, it may have been under-ascertained. BRCA1, BRCA2, and other hereditary cancer-related genetic test results were not available in the analytic dataset. The study therefore cannot estimate the prevalence of pathogenic genetic variants or determine their relationship with age, molecular subtype, treatment selection, or clinical outcomes. Future studies of young Saudi women should incorporate standardized genetic counseling and testing data whenever available.

Neoadjuvant-based systemic treatment was frequently documented, and mastectomy was more common than breast-conserving surgery. Studies from Jordan and Saudi Arabia have similarly documented substantial use of systemic therapy and surgery among young women with breast cancer [12,14,15]. However, the treatment variables reflected broad chart-derived categories rather than complete treatment pathways. Detailed systemic agents, dosing and schedules, sequencing, radiotherapy, treatment completion, surgical eligibility, genetic findings, patient preferences, and multidisciplinary decision-making were not consistently available. Therefore, the appropriateness or comparative effectiveness of the documented treatment approaches cannot be evaluated from these data.

No statistically significant differences between the two age groups were found in documented recurrence or death. These findings should be interpreted cautiously and must not be considered estimates of disease-free or overall survival. The analytic dataset did not contain complete dates of diagnosis, recurrence, last follow-up, or death; follow-up duration was not standardized; and subsequent outcomes were incomplete for patients who transferred care. A valid survival comparison would require time-to-event data, appropriate censoring, standardized follow-up, and adjustment for stage, tumor biology, and treatment. Accordingly, the recurrence and vital-status findings in this study are descriptive only.

From a clinical and public-health perspective, the predominance of symptomatic presentation and the documented occurrence of nodal, high-grade, and metastatic disease support prompt assessment of breast symptoms in younger women. However, this study did not evaluate diagnostic delay, screening effectiveness, or referral pathways and therefore cannot support changes to population-based screening policy.

The clearest health-system implication is the need for standardized documentation of symptom onset, diagnostic intervals, stage, molecular markers, genetic testing, treatment, and longitudinal outcomes. Linkage of multicenter clinical data with population-based cancer registries could help distinguish referral patterns from population epidemiology and inform service planning for younger women.

Strengths of this study include its focus on a group that remains underrepresented in the regional literature, inclusion of all eligible institutional records, transparent use of variable-specific denominators, and integrated reporting of demographic, clinicopathological, molecular, treatment, and recorded outcome data. Together, these features provide a useful institutional description of breast cancer in young and very young Saudi women and highlight areas for improved data capture and future multicenter research.

This study has several limitations. Its retrospective, single-center design creates potential selection and information bias and limits generalizability to the wider Saudi population. As a tertiary referral center, the institution may disproportionately receive patients with complex, advanced, or diagnostically challenging disease; this referral pattern may partly explain the documented proportions of grade 3 disease, axillary involvement, and distant metastasis. Missing documentation resulted in variable-specific denominators.

Complete AJCC/TNM stage, T4 features, and composite clinical or pathological stage were unavailable; consequently, tumor size, histological grade, documented axillary involvement, and distant metastasis cannot substitute for overall stage or support stage-adjusted interpretation. The dataset also lacked nationality or ethnicity, standardized diagnostic-interval data, detailed screening pathways, BRCA1/BRCA2 and other hereditary cancer-related genetic test results, detailed systemic treatment regimens and radiotherapy, and complete treatment and follow-up information. Some patients continued care outside the institution, further limiting the completeness of subsequent clinical and outcome information. Molecular subtypes were abstracted as documented rather than independently reclassified, and consistency of reporting criteria across the study period could not be verified.

The study did not include women aged > 40 years and therefore cannot establish whether younger women differ from the broader breast cancer population. Dichotomizing age at 35 years may have reduced statistical power and obscured continuous age-related trends. Multiple exploratory comparisons were conducted without global multiplicity adjustment, so statistically significant demographic findings should be interpreted as hypothesis-generating. Finally, the modest sample size and narrow age range may have limited the ability to detect clinically meaningful differences between the two age groups.

## 5. Conclusions

In this single-center descriptive cohort of women diagnosed with breast cancer at age ≤ 40 years, symptomatic presentation and potentially unfavorable documented pathological features were common. No statistically significant clinicopathological, molecular, or treatment differences were detected between women aged ≤ 35 and 36–40 years; however, these exploratory findings should be interpreted cautiously given the modest sample size and do not establish clinical equivalence. These findings support timely assessment of breast symptoms in younger women and highlight the need for standardized staging, hereditary-risk assessment, and longitudinal multicenter data to better characterize this population and inform service planning.

## Figures and Tables

**Table 1:** Demographic characteristics of the study cohort (*n* = 135).

Characteristic	*n* (%)
**Age, years, mean ± SD (range)**	34.0 ± 4.08 (22–40)
**Age group (*n* = 135)**	
≤35 years	75 (55.6)
36–40 years	60 (44.4)
**Marital status (*n* = 132)**	
Single	37 (28.0)
Married	84 (63.6)
Divorced	11 (8.3)
**Number of children (*n* = 122)**	
None	45 (36.9)
1–2	32 (26.2)
>2	45 (36.9)
**Residence (*n* = 134)**	
Riyadh	114 (85.1)
Outside Riyadh	20 (14.9)
**Parity status (*n* = 127)**	
Parous	82 (64.6)
Nulliparous	45 (35.4)
**Family history of breast cancer (*n* = 130)**	
No	92 (70.8)
Yes	38 (29.2)
**BMI, kg/m^2^, mean ± SD**	27.6 ± 6.12
**BMI category (*n* = 135)**	
Normal (18.5–24.9 kg/m^2^)	46 (34.1)
Overweight (25.0–29.9 kg/m^2^)	49 (36.3)
Obesity (≥30.0 kg/m^2^)	40 (29.6)

Note: Values are presented as *n* (%) unless otherwise stated. Percentages were calculated using available-case denominators. Missing observations were excluded on a variable-by-variable basis, and no imputation was performed. The denominator is shown when data were unavailable for one or more patients. BMI, body mass index; SD, standard deviation.

## Data Availability

The dataset used and analyzed during the current study is not publicly available because it contains sensitive clinical information and is subject to institutional and patient-privacy restrictions. A de-identified dataset may be available from the corresponding author upon reasonable request, subject to approval by King Saud University Medical City and the relevant ethical and data-governance authorities.

## Supplementary Materials

The following supporting information can be downloaded at: https://www.mdpi.com/article/10.3390/jcm15218293/s1, Figure S1: Distribution of age at diagnosis among the study cohort; Table S1: Availability and missingness of study variables in the full cohort; Table S2: Post hoc pairwise comparisons of BMI categories by age group.

## Abbreviations

The following abbreviations are used in this manuscript:

BMI	Body mass index
HER2	Human epidermal growth factor receptor 2
SD	Standard deviation
TNM	Tumor–Node–Metastasis
