# Supplementary material for: Clinicopathological Characteristics of Breast Cancer in Women Aged 40 Years or Younger in Saudi Arabia: A Single-Center Descriptive Study
Source: J Clin Med. 2026 Oct 24;15(21):8293. doi: 10.3390/jcm15218293 (PMC13764334; doi:10.3390/jcm15218293)
Supplement: Supplementary file 1 [file jcm-15-08293-s001.zip › jcm-4586622-supplementary.pdf]

Supplementary Materials

Clinicopathological Characteristics of Breast Cancer in Women Aged 40 Years or Younger in Saudi Arabia: A Single-Center Descriptive Study

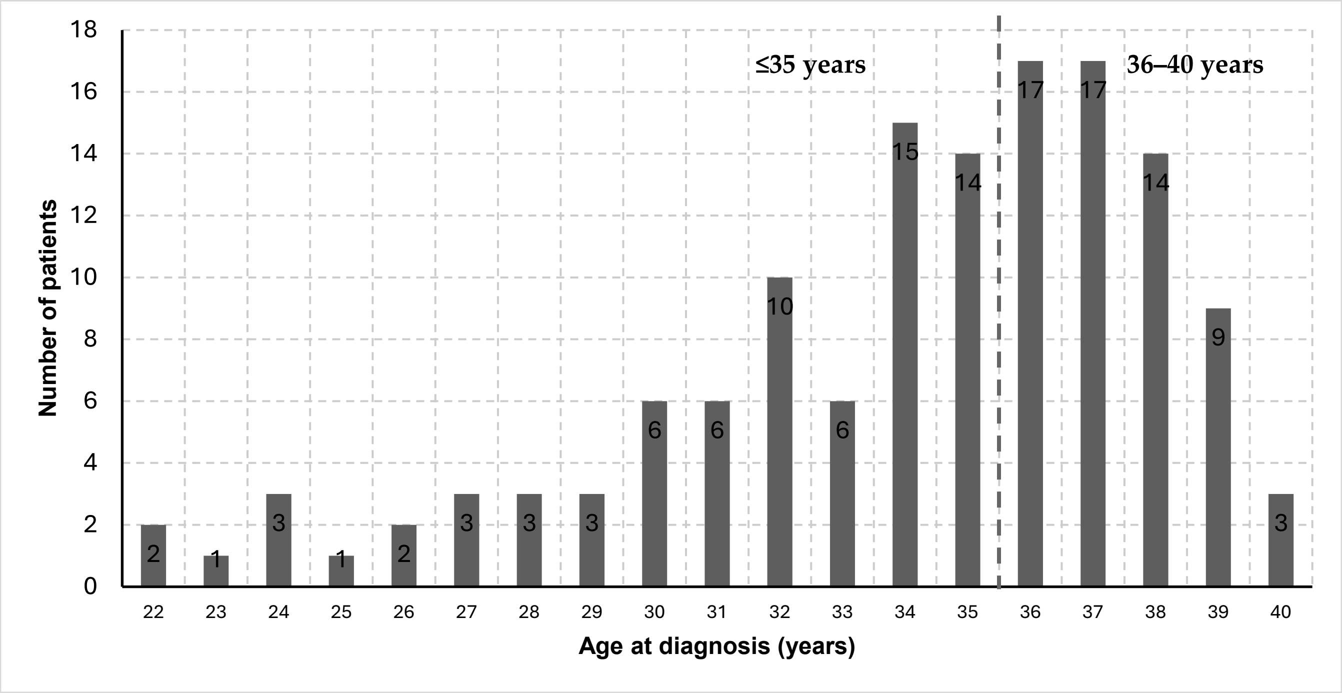

Figure S1. Distribution of age at diagnosis among the study cohort. Bars show the number of women at each single year of age. The 35-year cutoff was used for the exploratory age-group comparison.

Table S1. Availability and missingness of study variables in the full cohort (N = 135).

**Variable**	**Available, n**	**Missing, n**	**Missing, %**
Age	135	0	0.0
Marital status	132	3	2.2
Number of children	122	13	9.6
Residence	134	1	0.7
Parity status	127	8	5.9
Family history of breast cancer	130	5	3.7
Body mass index category	135	0	0.0
Histological diagnosis	135	0	0.0
Initial presentation	135	0	0.0
Tumor size	135	0	0.0
Axillary involvement	133	2	1.5
Distant metastasis	132	3	2.2
Histological grade	129	6	4.4
Documented molecular subtype	130	5	3.7
Primary treatment	135	0	0.0
Surgery	135	0	0.0
Recorded recurrence status	122	13	9.6
Vital status at last documented contact	120	15	11.1

Note: Availability and missingness were calculated from the variable-specific denominators reported in the manuscript. Transfer-of-care and undocumented outcomes were excluded from the corresponding recurrence and vital-status denominators.

Table S2. Post hoc pairwise comparisons of BMI categories by age group.

Pairwise comparison	Pearson χ^2	Unadjusted p	Holm-adjusted p	Interpretation
Normal weight vs obesity	7.516	0.006	0.018	Significant after Holm correction
Normal weight vs overweight	4.283	0.038	0.077	Not significant after correction
Overweight vs obesity	0.642	0.423	0.423	Not significant

Note: Pairwise 2 × 2 Pearson chi-square tests were calculated from the contingency-table cell frequencies reported in Table 3 after the significant overall BMI comparison. Holm adjustment was applied across the three pairwise tests. BMI, body mass index.
